# Understanding the barriers and facilitators to using self‐sampling packs for sexually transmitted infections and blood‐borne viruses: Thematic analyses for intervention optimization

**DOI:** 10.1111/bjhp.12617

**Published:** 2022-08-02

**Authors:** Paul Flowers, Gabriele Vojt, Maria Pothoulaki, Fiona Mapp, Melvina Woode Owusu, Claudia Estcourt, Jackie A. Cassell, John Saunders

**Affiliations:** ^1^ School of Psychological Sciences and Health University of Strathclyde Glasgow UK; ^2^ Department of Psychology Glasgow Caledonian University Glasgow UK; ^3^ Department of Infection & Population Health University College London London UK; ^4^ Department of Primary Care and Public Health University of Brighton Brighton UK

**Keywords:** barriers, blood‐borne viruses, facilitators, intervention optimization, qualitative, self‐sampling, sexual health, sexually transmitted infections

## Abstract

**Purpose:**

Self‐sampling packs for sexually transmitted infections (STIs) and blood‐borne viruses (BBVs) are widely offered. There are ongoing problems with reach and sample return rates. The packs have arisen without formal intervention development. This paper illustrates initial steps of an intervention optimization process to improve the packs.

**Methods:**

Eleven focus groups and seven interviews were conducted with convenience samples of patients recruited from sexual health clinics and members of the public (*n* = 56). To enable intervention optimization, firstly, we conducted an inductive appraisal of the behavioural system of using the pack to understand meaningful constituent behavioural domains. Subsequently, we conducted a thematic analysis of barriers and facilitators to enacting each sequential behavioural domain in preparation for future behaviour change wheel analysis.

**Results:**

Overall, we found that self‐sampling packs were acceptable. Participants understood their overall logic and value as a pragmatic intervention that simultaneously facilitated and reduced barriers to individuals being tested for STIs and BBVs. However, at the level of each behavioural domain (e.g., reading leaflets, returning samples) problems with the pack were identified, as well as a series of potential optimizations, which might widen the reach of self‐sampling and increase the return of viable samples.

**Conclusions:**

This paper provides an example of a pragmatic approach to optimizing an intervention already widely offered globally. The paper demonstrates the added value health psychological approaches offer; conceptualizing interventions in behavioural terms, pinpointing granular behavioural problems amenable for systematic further improvement.

**Statement of Contribution**

***What is already known on this subject?***

- Self‐sampling packs for sexually transmitted infections (STIs) and blood‐borne viruses (BBV) allow a person to take a series of their own samples and post them to a laboratory for testing.
- However, the use of self‐sampling packs for STIs and BBVs has been widely implemented without in‐depth assessment of user engagement or theorization.
- Some evidence suggests that the uptake of self‐sampling packs, and the return of samples to enable diagnosis, are socially patterned.
- Despite increasing and widespread use of self‐sampling packs across the United Kingdom, relatively little is currently known about their acceptability, or how they could be improved.

***What does this study add?***

- The thematic analyses show that self‐sampling packs offer a largely acceptable means to enabling STI and BBV testing and diagnosis; they remove many barriers to testing.
- Findings show that self‐sampling packs embody several modifiable barriers to use, reducing sample return and potentially amplifying health inequalities.
- This study presents a range of barriers and facilitators to the various behavioural domains included within the use of self‐sampling packs. It summarizes the findings ready for subsequent behaviour change wheel analyses.

## INTRODUCTION

The World Health Organization (WHO) estimates a global burden of 376 million newly diagnosed sexually transmitted infections (STIs), annually (World Health Organization, 2018). The WHO highlights a lack of progress in preventing STI transmission over time. Care of people with STIs and blood‐borne viruses (BBVs) is complex and improving outcomes is challenging; infections are often present without symptoms thus people may transmit the infection unknowingly. Equally, STIs and BBVs disproportionately affect some people, they are profoundly stigmatizing, and their prevalence is associated with typical health inequalities such as sexual identity, deprivation, non‐white ethnicity, geographic factors and younger age (Tanton et al., 2018; Woodhall et al., 2016). In response to increased disease burden across the whole population and reduced funding for STI care and prevention, novel models of sexual healthcare delivery have rapidly emerged without an evidence base.

In recent years, there have been attempts to use health psychology in the design of sexual health interventions. These have included behavioural interventions to improve condom use (Anstee et al., 2019; Stone et al., 2018); to enhance chlamydia testing (McDonagh et al., 2018, 2020); to promote HIV medication adherence (Spaan et al., 2020); to promote HIV testing (Flowers et al., 2019). There has been relatively less research on enhancing sexual health services. Exceptions being improving sexual health service use among university students (Cassidy et al., 2019), evaluating novel screening approaches (Footman et al., 2021) or the use of chatbots to provide sexual health advice (Nadarzynski et al., 2020).

Adding to this growing sexual health psychology, here we examine the provision of self‐sampling packs for STIs and BBVs. Typically, a person requests a self‐sampling pack online, which is delivered to their home. They then take their own samples (e.g., urine, genital swabs, finger prick blood), package the completed kits and post them to the laboratory for testing. Results are provided by text message/short message service (SMS), telephone or online. Alternatives include a person attending a sexual health clinic, taking their own samples on the premises and leaving their completed kit there. National guidance recommends a ‘full screen’ for people seeking STI testing as STIs often co‐exist and may be asymptomatic, which means that many infections would go undiagnosed without comprehensive screening. For this reason, when people request online testing, they are advised to test for chlamydia, gonorrhoea (swabs and or urine) in addition to HIV and syphilis (blood) – and they are sent a kit containing all appropriate components to do so. The return envelope has to comply with regulations for posting biological samples (e.g., urine/swabs/blood) and therefore has markings that state this on the outside. This is not amenable to change as we have to comply with these regulations.

This kind of home‐based self‐sampling and testing is becoming more commonplace across a range of health domains. The COVID‐19 pandemic has rapidly driven an increase in remote self‐care and the use of self‐sampling packs for COVID‐19 for PCR testing has become a central feature of attempts at COVID recovery (Department of Health and Social Care, 2021). Health psychology has much to offer in assisting with intervention development in these areas, for example, by either optimizing what is already available, or contributing to improved implementation and/or widening population reach.

Self‐sampling for STIs has been widely and rapidly adopted throughout the United Kingdom (UK). This has occurred as a pragmatic response to changes within the health care system and across wider communities of users (as increasingly life is digitally mediated). In some areas of England, it is intended to be the primary means of testing and diagnosing infections in asymptomatic people (Campbell & Marsh, 2017). This may be beneficial to many people who cannot easily access health facilities in person. However, evidence is emerging of unanticipated adverse consequences. Studies suggest that self‐sampling is underused by people who do not have post‐school education, older people, by men, by non‐heterosexual women and by racialized and minoritized people (Banerjee et al., 2018; Barnard et al., 2018; Kersaudy‐Rahib et al., 2017; Manavi & Hodson, 2017). Moreover, sample return rates range considerably, for example the National HIV Self‐sampling service in England reported return of kit rates varied between 49.6% in 2015 and 66.5% in 2019 (Public Health England, 2019a, 2019b).Treatment rates appear to be lower in individuals using self‐sampling than those attending sexual health services (Banerjee et al., 2018; Kersaudy‐Rahib et al., 2017; Manavi & Hodson, 2017).

In this paper, we report the first step of a multi‐staged intervention development process, which sought to optimize the use of self‐sampling packs. The analysis reported here was followed by a behaviour change wheel (Michie et al., 2011) analysis to specify potential intervention components that could enable optimization of the self‐sampling pack and its content (Flowers et al., 2022).

Although there is a growing literature regarding the process of intervention *development* (O'Cathain et al., 2019), the equally important process of intervention *optimization* is less well understood. While many key processes, and the use of health psychological tools and acumen are the same, divergence lies in considering the goals of the overall process. When *developing* an intervention, major practical concerns typically relate to acceptability, practicability and future implementability. These should be central to the overall process and demand the involvement of key stakeholders right from the start. In contrast, when *optimizing* an existing intervention, broad brush issues of acceptability and implementability should already have been established if the intervention is already available at scale. As a consequence, given the intervention is already established, there may be a more distinct focus on optimizing the pre‐existing intervention with new behaviour change content to extend the range of access and population reach to those most in need, for example, or to reduce inequalities and address emerging unintended negative intervention effects.

From a methodological perspective, increasingly intervention development in health psychology focuses on ‘behavioural diagnosis’ in which, within a given behavioural system, a single key fulcrum point for investing in behavioural analysis is chosen (Michie et al., 2014). By contrast in intervention optimization, a more holistic approach to understanding the behavioural system addressed by the intervention may usefully be taken, with a view to exploring and understanding how particular behaviours and their associated behavioural domains are connected. Where the interdependent behavioural elements have not already been set out in prior intervention development work, these may need to be specified post hoc, to form the focus of systematic optimization in order to ensure plausible points of optimization are clearly identified.

### Research questions

1. What are the behavioural domains of using self‐sampling packs for sexually transmitted infections and blood‐borne viruses that could form the focus of future intervention optimization?
2. What are barriers and facilitators to enacting each of the selected behavioural domains effectively?

## METHODS

### Participants

Participants were heterosexual people aged 18–30 years, and men who have sex with men (MSM) aged 18–65 years, the groups of people disproportionately affected by STIs in the UK. They included sexual health clinic attenders and a community sample (no requirement for experience of previous sexual health care was necessary to take part). Eleven focus groups and seven interviews were conducted between 2016 and 2017. To facilitate peer‐focussed discussion and maximize the expression of group norms, focus groups were run separately for MSM and heterosexuals. All participants were cisgender; just under half the sample were women. Sixty‐four percent (*n* = 36) were aged between 18 and 25 years. Most participants were of a ‘white British’ or ‘other white’ ethnic group (*n* = 40, 71%) with others reporting themselves as Black African, Indian Pakistani and of mixed ethnicity. Most reported ‘University’ as their highest level of education (*n* = 39, 70%). The majority identified as heterosexual (*n* = 40, 71%), and a quarter were MSM (*n* = 14, 25%). Those recruited from sexual health services (*n* = 20) all reported a recent STI, while of those recruited in the community (*n* = 36), one quarter (*n* = 9, 25%) reported to have ever had an STI.

### Settings

Sexual health clinics in London and Glasgow, UK. Community samples included work settings, stakeholder organizations and researchers' personal networks.

### Procedure

#### Recruitment sexual health clinic patients

Sexual health clinic staff discussed the study with eligible clinic attenders and asked them to complete an expression of interest if they were interested in participating. Expression of interest forms were paper‐based and clinic attenders placed them in a pad‐locked metal study box clearly labelled as LUSTRUM. The expression of interest form requested basic information from interested individuals (i.e., demographics and contact details) so that the research team could proceed with further contact. Only research team members had access to the locked box located in the clinic. Research team members visited the clinic on a daily basis to collect expression of interest forms and upon collection they were transported directly to the University building and locked in a filing cabinet. All collecting research members were fully trained in good clinical practice. Using the information on the expression of interest form, the researcher phoned the potential participant to discuss the study further and to coordinate participation as appropriate.

#### Recruitment community sample

Members of the public were recruited through purposive and convenience sampling, including snowballing, advertising on social media and through the research team's personal social networks. Posters, formal letters and/or emails were used to approach stakeholder organizations and diverse work settings to further widen recruitment. Interested parties completed an expression of interest form with brief contact details. The researcher phoned the potential participant to discuss the study further and to coordinate participation as appropriate.

#### Inclusion criteria

Eligibility for study participation included the ability to communicate in English and provide informed consent. Sexual health clinic attenders needed to have been diagnosed with an STI within the preceding six months. People in whom the STI was believed to be acquired as a result of sexual assault were not invited to participate to avoid potential additional psychological burden.

### Data collection

**FIGURE 1:**
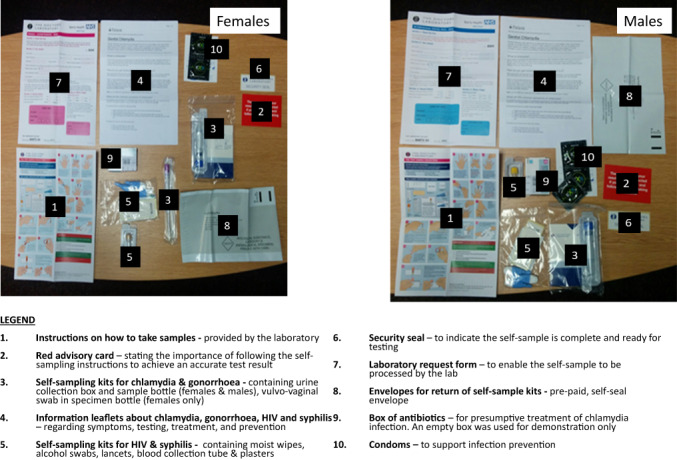
The self‐sampling packs used within the focus groups

The focus groups were guided by a topic guide and started with a demonstration of a comprehensive STI and BBV self‐sampling pack. As this study formed part of a programme of research into partner notification, the process of identifying, testing and treating sex partners of people with STIs (www.lustrum.org.uk), the pack also contained antibiotics. See Figure 1 for the pack contents used during the focus groups. In addition, participants were shown the partner notification pack packaging (envelope or small box, without branding or identifiable markings and which fits through standard letterboxes). Those using packs like this are strongly encouraged to use all the test kits as they would in a face‐to‐face interaction at a sexual health service. Where possible, discussions between focus group participants were encouraged rather than between facilitators and participants. Data collected were primarily concerned with understanding the barriers and facilitators to the use of the pack as a whole and its components. The topic guide structured flexible discussion around pack use and included the following broad questions: (1) What do you think would help someone use the pack? (2) What challenges might there be to using the pack? (3) What would enable you to use the pack? (4) What would stop you from using the pack? (5) How might a health advisor or a clinic help with pack use?

Participants were compensated for their time with a £30 retail voucher, redeemable in high street and online stores.

### Data analysis

Data were transcribed, anonymized and imported into NVivo (Version 10; Computer Software for Qualitative Data Analysis). Initially, one analyst (PF) led an inductive analysis exploring the data in relation to the behavioural system and associated behavioural domains related to the use of the self‐sampling packs. Subsequently, deductive analysis focussed a priori on the barriers and facilitators to each of the sequential steps identified by the initial inductive analysis. In this way, the analysis balanced an inductive understanding of how participants understood the distinct behavioural elements of the self‐sampling pack (e.g., system vs. domain vs. specific behaviours) and a deductive analysis of the barriers and facilitators to each of these distinct steps. One analyst (PF) led the deductive analysis of barriers and facilitators to each step. Two researchers (GV, MP) audited this analysis. Disagreements were resolved through discussion.

Where possible and appropriate, the data are presented as interactive exchanges illustrating the social nature of the focus groups. The selected extracts are representative of the underlying theme reflecting participants' collective perspectives. Within each extract P1, P2 etc. are used to illustrate the diverse participants within each focus group.

## RESULTS

### Research question 1. What are the behavioural domains of using self‐sampling packs for sexually transmitted infections and blood‐borne viruses that could form the focus of future intervention optimization?

**FIGURE 2:**
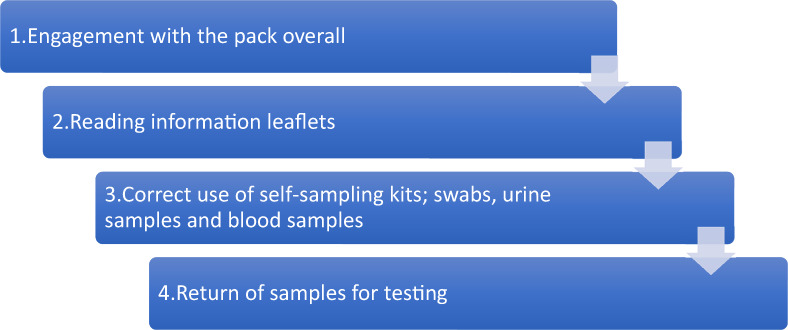
Inductively derived themes showing participants' perspectives of the sequential behavioural domains of using self‐sampling packs

Our inductive analysis highlighted four key sequential steps representing distinct behavioural domains that appeared meaningful to the participants, each consisting of a set of highly related specific behaviours. These are illustrated within Figure 2. Each step also presented a focal point for intervention optimization. Together, these behavioural domains and concomitant individual behaviours can be thought of as representing the overall behavioural system of self‐sampling pack use.

### Research question 2. What are the barriers and facilitators to enacting each of the selected behavioural domains effectively?

In relation to the second research question, results are structured according to the four key sequential steps associated with the overall behavioural system of using the self‐sampling pack.

#### Engagement with the pack overall

The pack, as a single entity, shapes the pack user's subsequent engagement with the overall pack contents and their use of the kits within it. As suggested in Figure 2, participants (P) understood and clearly valued the logic of the self‐sampling pack in discussions led by the facilitators (F). For example, in the extract below it is clear how the pack can moderate the impact of felt stigma associated with using sexual health clinics:

P4 I think the pack is very convenient, […..] I think anybody would feel very uncomfortable going into the clinic and getting it [STI testing] done, reason why I say it's convenient because you can do it in your own space, not to say that nobody's up there to harm you or laugh at you doing anything but it's just much more comfortable and convenient for the person themselves because it is quite embarrassing, I mean personally speaking it is very embarrassing but…

F1 Do you mean going to the clinic?

P4 Going into the clinic and, you know, especially if you know what you are going in for, so, hence why I think it's, I think it's actually incredible to be honest. I think it's very, very convenient, and yeah.

(Heterosexual women, patients, London, focus group)

However, beyond general appreciation for the logic of the overall pack, participants almost universally identified a series of barriers to engagement with it. Almost all participants felt that the pack overall was confusing to potential users. Participants talked about being overwhelmed when they first looked at it: ‘It just exploded in for me and I don't really know where to start’ (Heterosexual man, public, Glasgow, focus group). Participants suggested ways of ameliorating this, clearly stating that there should be guidance explaining the structure and contents of the pack. These overall ‘pack‐level’ instructions should include clear visuals and simple messages highlighting exactly what to do with the pack and its varied contents and the order in which to do them.

Study participants recognized that pack users' engagement with the pack may well take place in stressful circumstances; particularly at times when there is a high chance of infection. High stress levels could negatively shape attention and decision‐making processes relating to self‐sampling kit use. Adding to the strong feelings about pack design, participants also raised further issues about the life circumstances of some pack users, which could impact upon pack use. Participants outlined the ways such circumstances might amplify some of the problematic elements of pack design. Participants believed that the re‐organization and a new pack structure might also be important as it had a potential role in reducing stress levels and enabling compliance with specific instructions for the self‐sampling kits within it. The potential age of pack users, their disabilities or domestic circumstances were understood to be important in relation to using the pack ‘It's a mess, if you live in shared accommodation or with family and you want to keep it secret or something, it would be hard to stuff all this away, if Mum comes upstairs’ (Heterosexual man, public, London, focus group). Participants also suggested the contents of the kit could be presented within a clearer physical framework, variously referred to as an ‘airline meal’, ‘gadget‐packaging’, ‘a chocolate box’ or a ‘graze box’™ in which individual components are packed within a stable card/plastic structure, which allows each one to be seen. This attention to *how* to present the contents of the pack overall did not relate to aesthetics but to pack usability. A well‐designed pack could enhance pack user self‐efficacy, minimize perceived risks of non‐compliance and, for a minority, indicate the degree of professional *care* in the absence of a face‐to‐face interaction. Participants also suggested the potential of having online video support that could not only assist with the use of the pack overall but also provide indepth information about STIs and BBVs and their health consequences.

#### Reading information leaflets

Participants outlined several barriers and facilitators to reading the leaflets within the pack itself, highlighting the importance of literacy and health literacy levels and the ability to retain attention. Additionally, the particular challenges of engaging with the leaflets among non‐native English speakers, those with dyslexia or learning difficulties were stressed. The extract below, for example shows how important the leaflets could be in shaping pack users' understandings of the consequences of the infection. In turn, this could be important for wider engagement with treatment and self‐sampling:

F2 You said earlier on that it was really important that people actually understood what sexually transmitted infections can do to the body.

P1 Aye …the information sheet?

F2 Aye.

P1 This is the same one I got shown.

F2 So, see as part of the pack that's exactly what you get, do you think that's, I mean do you find that useful?

P1 Sometimes it can, looking at it, it could be too much for somebody to read. Just break it down into detail, like wee [small], short sentences that physically somebody could understand. Whereas, you might get somebody that will just read halfway through it, and just go, ‘Oh do you know what……Get that away from me!’. [……] Think about it… if you shorten it down to two pages, short and sweet.

F2 So, essentially re‐structure this, make it shorter and I think you said just keep it to the essentials, make it easier to read?

P1 Aye.

(Heterosexual man, patients, Glasgow, focus group)

Participants agreed that breaking the text up into smaller units and the use of better visuals may enable the pack users to better engage with self‐sampling kits:

P3 Aha, yeah. Like a small thing and it's all in little paragraphs kind of thing. I think that those little leaflets are really easy to read, and I'm quite happy to just sit and read one of them in my own time. Whereas this kind of looks like a bank statement to me a little bit and I'm a little bit like…no thank you. It just looks like something that my mum and dad would get in the post, not something that I would sit and read, if that makes sense.

F2 What's the difference between your mum and dad and you? Can you be a bit more specific?

P3 Well, it looks really formal, like something that you would receive, like a bill or something in the post. Just the way it's all laid out.

(Heterosexual women, patients, Glasgow, focus group)

Participants also highlighted the potential to improve the leaflets further to include a statement on how long the self‐sampling process would take to complete.

#### Correct use of self‐sampling kits; swabs, urine samples and blood samples

For many of the participants, the self‐sampling kits within the pack were seen as relatively unproblematic and straightforward. However, one participant struggled with the idea of self‐sampling. For her, a key barrier to self‐sampling related to how she perceived the professional roles and responsibilities of clinical staff:

Why am I doing the doctor's job? Why am I taking my own test? I'm sorry, I'm pure getting annoyed if you're telling me to do my own…sorry. […] What if I do it wrong and then it comes back inconclusive and then there's…wasted even more money. Or, like, goes back saying that I don't have it when actually I do have it.

(Heterosexual women, public, London, focus group)

Such worries over the accuracy of the test results following self‐sampling were expressed by several participants across different focus groups. More broadly, engagement with the pack's multiple self‐sampling kits, for a variety of STIs and BBVs, was seen by some as a convenient and time efficient way to check their wider sexual health. Perhaps the biggest barrier to complying with the various self‐sampling kits was the inclusion of the HIV self‐sampling kit within the pack. This was understood to be problematic for a few reasons: the particular stigma associated with HIV; the perceived gravity of an HIV positive result; and challenges with blood‐based sample collection such as fear of needles, or perceptions of not being able to collect a viable sample of blood.

P3 And I suppose the emotional stress as well that you are going through, or that I would be going through, thinking, have I potentially got an STI? And then trying to do a pack like that at home, I would probably find that quite stressful. Like,

I'd

already be stressed and then

I'd

be like, ‘How the hell do you use this f******** thing?’ And then

I'd

be like, ‘Bastard!’ [voices overlap]…

[General laughter].

P3 It stressed me out [voices overlap]

P1 Just…[……] can I just add as well, I think it was pure, like, ‘Aye! We're going to test you for HIV!’. I do not know about anybody else, but if that was me, I'd be like, ‘What the f*** are you talking about HIV? I'd be like, I thought I had chlamydia. I know, like, you do that ‘cause if you are pregnant, they do give that test and all that as well, like…what is it, like, hep C and all that…

P2 Hepatitis.

P1 Aye, ‘cause I even…I knew I didn't have it, but when I was waiting on those results, I was like, I hope I don't have it. I hope I don't have it. I just…it's one of those things, know what I mean. Imagine hearing that on the phone. You'd be like, ‘Why are you testing me for HIV? I thought it was just chlamydia.’

P3 So maybe just focus on, like, the problem in hand…

P1 The problem in hand, yeah.

P3 …and not necessarily talking about other bigger things if you were already feeling a bit stressed, aye.

(Heterosexual women, public, Glasgow, focus group)

One group of heterosexual men talked about how the inclusion of the HIV self‐sampling kit could actually detract from the use of chlamydia self‐sampling kits.

F2 Right, basically not do the tests?

P3 Not do the tests. Because if I do the Chlamydia test and send it, where is the accompanying HIV test…which I, I'd hate to do.

F2 So basically, a lot more information and perhaps assurances for why…

P3 Assurances for why it should be done, reasons why those tests need to be done; you cannot just find out about this and leave the other…because they are kind of all in a group.

P1 Ignorance is bliss.

P3 Yeah. [Laughter].

(Heterosexual men, public, London, focus group)

However, participants also talked of ways of minimizing these barriers to chlamydia self‐sampling through making sure there was clarity concerning the choice of completing chlamydia self‐sampling but not the HIV self‐sample. Echoing the earlier findings, the sense of choice in selecting some, but not all, of the self‐sampling kits could be facilitated through attending to the structural elements of the pack itself.

#### Return of samples for testing

The overall appearance of the sample return envelope and its contents raised some concerns for participants. These related primarily to the perceived stigma of STIs and BBVs and concomitant perceptions that postal staff may not handle the return envelope correctly. Participants also outlined some barriers in relation to perceptions of the safety and effectiveness of the postal delivery system in enabling samples to get to the laboratory within the timeframe needed for samples to remain viable for testing. The extract below suggests a simple but effective solution to this perceived problem:

P5 Do you get a notification when your tests arrive, your kit arrives at the…? I think that would be my big, I know that you have to wait anyway but…

P1 I never had one.

P5 That's always been my concern about these things is like, I know that…

P1 Has it got lost in the post? Has someone got a pot of my urine?

P5 I know that's potentially something that is almost, is it always going to be, is it two to four weeks, or whatever it is, you know, you do not know but if it was anything that could.

F2 Delivery receipt to say that it's been – no, is that what you mean?

P5 Yeah, yeah.

F2 A delivery receipt to know that it's gone to the right person.

P5 That would be, even if it was just you know, when it gets to the lab, they send you a text.

P2 Yeah.

(MSM, public, London, focus group)

Participants outlined how some of these concerns relating to the perceived efficacy of the postal system could be easily remedied through the provision of a range of return options, such as dropping off samples to a sexual health clinic, avoiding queues within clinics by using a ‘drop box’ facility where completed self‐samples could be safely returned to a clinic and then a lab. Participants acknowledged that the relative pros and cons of such an approach were moderated by the setting of the clinic, with those in more rural and small communities probably preferring postal routes rather than the expense, potential social exposure and felt stigma that might occur through using clinical contexts:

P3 Conversely, I think for some people, the ability to just go and drop it in a post box is very appealing as well, as opposed to like if you live in a small town, you know, [NAME OF TOWN] is a bigger town, you…

P1 Or having to wait in a queue or something like that, you know.

P3 Yeah. The waiting room, of course, is full of your close friends, family and colleagues, as you drop off your sample, they are all dropping off theirs.

P4 Yeah, people in rural areas you'd imagine it could be very important.

(Heterosexual men, public, Glasgow, focus group)

Table 1 (below) summarizes the findings of the thematic analysis. The column on the left shows the key sequential step from Figure 2.

**TABLE 1:** A summary of the key barriers and facilitators associated with use of the pack (key stages detailed in Figure 2)

Key elements of pack use (from Figure 2)	Barriers	Facilitators
1. Engagement with the pack overall	For the pack user, the pack as a single entity can be overwhelming and associations with potential STI diagnosis and consequences can be off‐putting.	Pack users' understanding of the relative ease of using the pack when compared to visiting the clinic can enhance engagement and increase motivation with pack use overall.
	The emotional impact of potential STI diagnosis may distract the pack user from using the pack and attending to instructions about using the pack.	For the pack user, a weighing‐up of the stigma and embarrassment associated with visiting the clinic compared to using the pack can facilitate pack use.
	The different perceptions about, and sampling methods for, chlamydia, gonorrhoea and HIV can put users off engaging with the pack.	Inclusion of pack‐level instructions could support engagement with the pack and all its contents.
	Once opened the pack contents can look chaotic and deter engagement with the pack contents.	A clear physical framework that organized the pack contents could support engagement with the pack minimizing feelings of being overwhelmed.
	Pack‐related stress (feeling overwhelmed with the volume and complexity of pack content) can amplify STI‐related stress and reduce attention and engagement with the pack.	A compartmentalized designed pack that systematically supports pack users in a step by step, simple approach could support engagement with the pack.
	Concerns about perceptions of ability to use the pack contents correctly can put the pack users off using the pack entirely.	Links to internet sites which provide further details of the pack and videos and how to use it would enhance pack use.
	The individual circumstances of pack users might add specific problems with pack use overall (e.g., young person living with parents or sharing accommodation).	Detailed, easily accessible, online support might assist with pack use.
2. Reading information leaflets	Too much text can reduce attention and engagement with the leaflets that provide direction and support for pack users.	Articulation of the potential health impact of STIs and BBV early on within the leaflets may motivate pack users to continue to read the leaflets and engage with the pack.
	For the pack user an overload of textual information can create problems with attention and focus and lead to further feelings of being overwhelmed.	Simplified clear visual communication, visual aids and short text extracts would aid the pack user reading and engaging with the leaflets.
	For pack users, variations in literacy levels may systematically reduce engagement with the leaflets and lead to inequalities in pack use with only those with higher literacy levels using the pack and its kits.	A sense of the time needed to complete each specimen collection should be clearly articulated and may enhance effective use of the pack.
	Engaging with the leaflets may be difficult for pack users whose first language is not English adding to the stratification of pack use.	Key information should be clearly presented to enhance use of the pack and the kits within it.
3. Correct use of self‐sampling kits; swabs, urine samples and blood samples	Pack users are concerned about their ability to collect samples correctly and that this may negatively impact on the accuracy of later results.	A clear and full explanation of the full range of self‐sampling kits (including HIV) may encourage uptake and compliance with kit use.
	Pack users may be put off from using *any of* the components of the pack by the inclusion of the HIV self‐sample kit.	Providing a sense of choice and partial uptake of the self‐samples (HIV or Chlamydia) may enhance pack users' uptake of self‐sampling for one or all infections (but may not be clinically desirable).
	Pack users' beliefs that they will struggle with collecting blood samples may discourage uptake of these specimens.	
4. Return of samples for testing	Pack users' concerns about the appearance of the return envelope may reduce willingness to return samples.	Providing a range of return options may increase pack users' willingness to return samples.
	Pack users' perceptions that the appearance of the pack may be read as a diagnosis of an STI may reduce willingness to return samples.	A receipt notification may increase pack users' willingness to return samples.
	Pack users' concerns relating to safety and effectiveness of the postal system may reduce willingness to return samples.	
	Pack users' perceptions that the returned pack might be unsealed or damaged in the post may reduce willingness to return samples.	

## DISCUSSION

To our knowledge, this is the first study to systematically explore the optimization of self‐sampling packs for infectious disease. We have drawn on a behavioural lens to conceptualize this widely used intervention in a way that enabled us to lay the foundations for the subsequent process of intervention optimization. Using qualitative data from diverse samples of patients and the public, we have used both inductive and deductive thematic analysis to detail that although the use of the self‐sampling packs can be thought of as a single activity it makes sense to understand it as a behavioural system. This system is made of diverse yet interdependent sequential behavioural domains. This envisioning of the self‐sampling pack as a behavioural system is important because we found that there were distinct barriers and facilitators to engaging with the various diverse behavioural domains within the pack system overall. When offered the opportunity, our participants could quickly offer insights into how these packs could be improved and could clearly detail problems with their use that could readily be addressed. Resolving issues with pack use early within this chain of sequential behaviours could have ‘cascade’ effects on other subsequent behavioural elements enabling more people to use the pack and all of its contents rather than risking attrition or partial use.

Our analyses show how initial consideration of the pack design and make‐up overall may be an important first step to widen access and increase use. The volume and organization of pack contents were perceived to be a major barrier to some pack users. Equally, in relation to the content of the packs, our analysis highlighted particular problems with the instructions for the different self‐sampling kits needed to test for a range of STIs and BBVs; these were too detailed and too wordy and could be improved with relatively simple adjustments to the formatting and presentation of kit instructions. In a parallel project with a sample of participants with mild learning difficulties, we found broadly similar results (Middleton et al., 2021). We found that within the behavioural domain of the ‘Correct use of self‐sampling kits’, some of our participants differentiated the HIV self‐sampling from the other self‐sampling kits. Finally, our analysis showed that there were some problems with the return of the samples that potentially could be removed with minor adjustments to the process.

Our analyses focus on the proximal barriers and facilitators to self‐sample pack use and do not address the more distal barriers and facilitators to sexual health services per se. These do intersect with the use of self‐sampling packs. Major barriers to sexual health service use in general include knowledge of sexual health services (Ayon et al., 2018; Ma et al., 2017), feelings of embarrassment (Fleming et al., 2020), the perceived and felt stigma of sexual health service use (Fleming et al., 2020; Ma et al., 2017; Parchem & Molock, 2021), concerns over the flow and safety of information (Ayon et al., 2018; Fisher et al., 2018; Parchem & Molock, 2021) and perceptions of likely inequitable treatment for some populations (Fisher et al., 2018).

Equally, our findings suggest that individual, user‐level problems with the pack may well have medium and long‐term impacts that amplify existing health inequalities. Sexual ill‐health itself is patterned by classic markers of health inequalities such as socio‐economic status and ethnicity, in addition to particular intersections with sexuality and age (Public Health England, 2019a, 2019b; Sonnenberg et al., 2013). Although not the direct focus of our work here, it also seems likely that the problems with pack use that we have identified are also patterned along the same lines. In this way, those most vulnerable to sexual ill‐health may also be those least likely to benefit from interventions such as self‐sampling packs. As the COVID‐19 pandemic has forced further innovation and reliance on telehealth and remote self‐management, it may be that those with the greatest need are also those that are particularly underserved. It is interesting to consider how these same dynamics may be equally important for self‐sampling for COVID‐19 for PCR tests or self‐testing for COVID‐19 with lateral flow devices or indeed the vast range of other health conditions in which self‐sampling or self‐testing may be useful.

Our approach to understanding the behavioural system of self‐sampling pack use and the range of interconnected behavioural domains is vital for executing the subsequent steps of intervention optimization effectively. These analyses are reported elsewhere (Flowers et al., 2022). We would suggest that current guidance on how to select behaviours to target within intervention development often underestimates the complexity and interdependencies of the elements of behavioural systems (Michie et al., 2014). Within the current project, and its focus on intervention optimization, we sought to have a distinct inductive analytic focus on understanding the behavioural aspects of the use of self‐sampling packs from the pack user's perspective. Overall, we believe that our approach to pragmatically identifying meaningful behavioural domains worked well. This step of conceptualizing the behavioural foci involved in the intervention that we have optimized was necessary to facilitate the more deductive analyses, matching appropriate barriers and facilitators to specific behavioural domains and concomitant behaviours. In turn, this was essential to enable the delivery of the subsequent behaviour change wheel analyses that followed (Flowers et al., 2022). We believe that deep and vital insights into behavioural systems to assist with the choice of behavioural target for behaviour change interventions can be gained from inductive qualitative research. Therefore, further research in this area is needed. Systematic exploration of how patients and health care providers understand the behavioural system that is at the heart of a behavioural diagnosis should be conducted. Diverse approaches to data collection and data analysis may enhance the field. These could include exploring ways of usefully visualizing behavioural systems and focussing inductively on how different stakeholders (e.g., patients and providers describe, conceptualize and understand a behavioural system).

In relation to limitations, although we tried to recruit a highly diverse sample able to illuminate the social patterning of self‐sampling pack use, the actual sample had particular biases. For example, all participants were cisgender, overall they had high educational attainment levels, and they all spoke English well. We had little representation from the upper part of our age range. It is worth noting that the nature of qualitative research and the use of focus groups in particular can partially compensate for these biases to some extent. Participants can, and do, adopt the perspective of the ‘other’, rather than focus on their own experience. However, we believe future, more targeted qualitative work could directly address self‐sampling pack use for STIs and other conditions with sampling strategies that speak more directly to those populations that experience the most problems in use or the lowest return rates of samples. Further, the analysis reported here is limited by its sole reliance on qualitative methods. If it had been possible within the budget and programme timeline, we would have complemented our qualitative assessment of barriers and facilitators to pack use reported here with additional quantitative assessment. Further complementary approaches could have focussed on ‘think aloud’ explorations of pack use (Jaspers et al., 2004). This could have brought an additional perspective to understanding barriers and facilitators. Furthermore, the bulk of the analysis was conducted by one experienced analyst rather than coded by a wider group of researchers within the team. Along these lines, it is hard to appraise the relative importance of each barrier or facilitator in any objective manner. However, at a later stage within the whole intervention optimization process, we used the APEASE criteria (Michie et al., 2014) with a group of mixed health professionals to address eventual recommendations in terms of Acceptability, Practicability, Effectiveness/cost‐effectiveness, Affordability, Safety/side‐effects and Equity. In this way, the overall project provided a number of safety checks to reduce the risks associated with our approach and the reliance on qualitative data analysis. In addition, the context in which our data were collected potentially shapes the value of our findings. We collected the data as part of a wider intervention optimization process of a partner notification intervention (Estcourt et al., 2020). As such, the data were collected in the context of a study where participants were primed by our research procedures to consider situations in which people who used the packs were at a high chance of having acquired an STI. Moreover, when packs were discussed, they contained antibiotic treatment for chlamydia, which is not current standard of care.

## CONCLUSION

This qualitative study represents the first step of research within a larger project concerned with optimizing self‐sampling packs for STIs and BBVs. This study provides a novel exemplar of how to use qualitative data and both inductive and deductive thematic analysis to explore the factors shaping a series of interrelated behavioural domains concerning a widely used intervention. Engaging public and patients in this way led to very useful insights concerning ways to optimize the pack and potentially increase sample returns. Through thematic analyses, we have shown that use of the pack overall was sometimes experienced as overwhelming, that pack‐level instructions were challenging to many, and that the poor overall pack‐usability was likely to reduce pack use. These insights formed the basis of further analysis to specify ways of optimizing the packs in greater detail. Finally, although our analysis is concerned with the process of optimizing self‐sampling packs for STIs and BBVs, we believe there are many commonalities in relation to considering ways to optimize self‐sampling for a range of other conditions.

## AUTHOR CONTRIBUTIONS

**Paul Flowers:** Conceptualization; formal analysis; funding acquisition; methodology; supervision; writing – original draft; writing – review and editing. **Gabriele Vojt:** Formal analysis; investigation; validation; writing – review and editing. **Maria Pothoulaki:** Formal analysis; investigation; validation; writing – review and editing. **Fiona Mapp:** Investigation; writing – review and editing. **Melvina Woode Owusu:** Investigation; writing – review and editing. **Claudia Estcourt:** Conceptualization; funding acquisition; writing – review and editing. **Jackie Cassell:** Conceptualization; funding acquisition; writing – review and editing. **John Saunders:** Conceptualization; funding acquisition; writing – review and editing.

## Funding information

This work presents independent research funded by the National Institute for Health Research (NIHR) under its Programme Grants for Applied Research Programme (reference number RP‐PG‐0614‐20009).

## CONFLICT OF INTEREST

None.

## ETHICAL APPROVAL

Ethical approval from Glasgow Caledonian University Research Ethics Committee (HLS/PSWAHS/A15/256) and NHS Ethics Approval (16/NI/0211) were obtained.

## DISCLAIMER

The views expressed are those of the author(s) and not necessarily those of the NHS, the NIHR or the Department of Health. The funders had no role in study design, collection, management, analysis and interpretation of data; writing of the report and the decision to submit the report for publication.

## Data Availability

Due to the qualitative nature of this research, supporting data such as full interviews with participants are not available to the public.
